# Establishing Operational Descriptive Definitions for Neurologic Abnormalities Identified During Gaiting in Dogs

**DOI:** 10.3390/ani16142144

**Published:** 2026-07-10

**Authors:** Rodney S. Bagley

**Affiliations:** Department of Veterinary Clinical Sciences, College of Veterinary Medicine, Iowa State University, Ames, IA 50011, USA; rsbagley@iastate.edu

**Keywords:** medical terminology, dogs, gait abnormalities, neurologic

## Abstract

Accurate identification of gait abnormalities is fundamental to clinical evaluation in both veterinary medicine and translational research. Commonly used descriptors such as ataxia, paresis, hypermetria, and lameness are frequently applied as single-word summaries of complex movement disorders, yet these terms lack precise, universally accepted operational definitions. Dictionary definitions are often generic or ambiguous, leading to variability in interpretation among clinicians, students, and researchers. This review evaluates commonly used neurologic and gait-related terminology in dogs, examining historical origins, denotation and connotation, as well as the limitations of current usage in a clinical setting. For each term, clinically observable features are delineated to establish functional, operational definitions grounded in movement analysis and neurophysiologic processes. The goal of this reappraisal is to enhance clarity, consistency, and diagnostic accuracy in veterinary neurology by establishing standardized descriptors for gait abnormalities in clinical practice, teaching, and research.

## Simple Summary

When describing patient abnormalities, it is critical that the clinician uses appropriate terminology that is universally agreed upon. This avoids miscommunication among colleagues about patient abnormalities noted during physical assessment. Unfortunately, while numerous patient descriptive terms have common use connotations, there remains a lack of agreement on the clinical expression of clinical abnormalities, making consistent assessments difficult. The common-use terms ataxia, dysmetria, spasticity, lameness, and paresis are discussed in this review to motivate a formal effort to provide specific clinical descriptions for these terms. Additionally, clarification is provided for the terms upper motor neuron and lower motor neuron with respect to use with clinical signs. Denotation and connotation of word choices are provided, as well as clinical descriptive features, allowing for a reappraisal of terminology usage in clinical practice, research, and clinical teaching when describing neurologic signs affecting gait in dogs.

## 1. Introduction

A cornerstone principle of clinical medicine is patient evaluation. The clinician’s role in this process is being an observer, describer, and documenter. Language is the functional tool for documenting clinical observations and manifestations of disease states. In the modern, complex, increasingly specialized, and compartmentalized medical delivery environment, technically accurate medical communication is an essential clinical skill influencing patient evaluation and management. Similarly, assessment of subject responses in clinical trials and evidence-based medical science must accurately document patient assessments for validation of clinical outcomes. Standardizing and recording medical information were the motivations for the problem-oriented medical record including the SOAP (subjective, objective, assessment, plan) format [1]. In addition to documentation, communicating medical aspects of a patient’s condition through various channels reflects the thought processing of the attending medical provider [1]. To ensure consistent and reliable communication of clinical findings, medical terminology must be technically accurate and supported by a universal, unambiguous lexicon. In this review we will consider familiar examples of word choices used to describe abnormalities of gait-related movements that are inherently ambiguous, incomplete, outdated, and/or confusing. The words discussed are entrenched in veterinary semantics and have significant influence on how clinical thinking is recorded and communicated to students and colleagues.

For this review, each word/phraseology choice includes a standard dictionary definition (denotation) provided from a representative dictionary (Merriam-Webster-Medical Dictionary (MWM) and Merriam-Webster Dictionary (MW)) as well as word derivations (historical context) [2,3]. In addition, clinically observable features of these abnormalities are provided to expand the clinical meaning of these terms. The terms “upper motor neuron” and “lower motor neuron”, “ataxia”, “hypermetria”, “lameness”, and “paresis” are discussed as, surprisingly, there is no scientific consensus for definitions or clinical connotations of these words. This discussion is not intended to encompass all word choices requiring refinement and reappraisal, but rather frequently used words and phrases during clinical patient evaluation that require operational definitions for clinical evaluation. Ultimately, these efforts should improve technical accuracy in documenting and communicating clinical manifestations of disease during medical observation, clinical teaching, and translational clinical science.

## 2. Terms Used to Describe Functional and Physiologic Concepts

### 2.1. Upper and Lower Motor Neuron

The terms upper motor neuron (UMN) and lower motor neuron (LMN) are regularly used to describe the location of an anatomical abnormality and/or a physiological (functional) process within the nervous system. The origin of these terms is attributed to Gowers in 1886 in the text “A Manual of Diseases of the Nervous System” and subsequent editions published in the late nineteenth and early twentieth century [4,5,6,7,8]. Gowers purportedly developed the term “upper motor neurons” to indicate neurons with cell bodies located in the motor cortex that extend axons to the medulla oblongata (expanded to include brain stem nuclei) and the spinal cord. “Lower motor neurons” are neurons with cell bodies located in the spinal cord (ventral gray matter) as well as brain stem nuclei which exit the central nervous system and directly innervate skeletal muscles.

Gower’s original interpretation was related to the motor functionality of the nervous system and, specifically, the pyramidal (corticospinal) system influence on a motor neuron located within the ventral gray matter of the spinal cord. The word choice of “motor” in both upper motor neuron and lower motor neuron clearly implies that the neurons under discussion are associated with motor functions of the nervous system. In all three editions there is a sentence that discusses the conceptualization of a central controlling motor component of the nervous system and depicts a two-order neuron connection.

“*On the hypothesis that I have advanced, this control is exerted by the termination of the pyramidal fibres, i.e., of the upper motor segment, by the structures degeneration of which probably causes spastic paraplegia*.”

In the Third Edition (Figure 74 on page 213), there is a drawing that depicts the anatomical and functional relationship of a central controlling neuron terminating on an anterior horn cell of the spinal cord [4,5,6,7,8].

Upper motor neuron and lower motor neuron used as anatomical and physiological concepts of nervous system organization are acknowledged as a fundamental relationship in the understanding of nervous system function. We have faithfully adopted these terms from human medicine and accepted this terminology into common veterinary professional practice. When this terminology entered the veterinary lexicon is uncertain; however, these terms are clearly described in Dr. De Lahunta’s first edition “Veterinary Neuroanatomy and Clinical Neurology” [9]. These same terms are also found in various, but not all, veterinary neurology textbooks in this same era [10,11,12,13,14,15,16]. Dr. De Lahunta used the term upper motor neuron as the “*motor system confined to the central nervous system that is responsible for the initiation of voluntary movement*” [9]. Relative to the lower motor neuron Dr. De Lahunta stated “*This is the efferent neuron of the peripheral nervous system that connects the central nervous system with the muscle to be innervated.*” Dr. A. S. King described the lower motor neuron as “*an alternative name for the alpha (skeletomotor) neuron*” [14]. In McGrath’s text “Neurologic Examination of the Dog”, the phrase “lower motor neuron” is stated relative to a discussion of “motor cells” of the “ventral gray horn” [15,16].

Based on the original conceptualization and terminology, it would seem most appropriate to apply these terms only when a disease process specifically involves neuronal populations with predominantly motor functions. However, although these terms remain deeply rooted in veterinary neurology, their use has expanded beyond their original neurophysiological intent and now commonly includes descriptions of ***clinical signs*** as being “UMN” or “LMN” in character. This application is problematic for several reasons:

1. **Directional terminology (“upper,” “lower”) is anatomically imprecise.** Anatomical nomenclature should rely on standardized directional terms such as cranial, caudal, rostral, proximal, and distal, rather than colloquial spatial descriptors.
2. **Clinical signs attributed to “UMN” or “LMN” dysfunction often reflect mixed pathology.** Structural diseases of the spinal cord or peripheral nerves commonly affect multiple neuronal populations simultaneously.
3. **“UMN/LMN” terminology used as “signs” does not account for sensory involvement.** Many neurologic diseases affect both sensory and motor pathways. Describing clinical signs as “UMN” or “LMN” implicitly suggests a motor-specific dysfunction, even when sensory deficits are equal or more prominent.

First, the descriptors “upper” and “lower” are imprecise within anatomical nomenclature and do not accurately convey spatial relationships within the nervous system [2,3]. The terms “up” and “upper” are colloquial directional expressions used to indicate the relative position within the body. Although useful in everyday language, such terms are not anatomically specific and should be avoided in contemporary medical communication.

The terms up, upper, and lower should be replaced by standard anatomical terminology that conveys precise spatial relationships. Descriptors such as cranial, caudal, rostral, proximal, and distal provide anatomically accurate localization [17]. Similarly, spinal cord pathways are more accurately described as projecting cranially or caudally rather than as “ascending” or “descending,” thereby maintaining consistency with accepted anatomical nomenclature.

Second, relative to the specific terms “upper motor neuron” and “lower motor neuron” used as operational terms, the actual phrases “upper motor neuron” and “lower motor neuron” are not found as explicit phraseology in Gowers’ original texts as clinical descriptors of patient signs. There is a single mention of an “upper motor segment” in the first three editions of the text. There is no statement that these terms are associated with a specific type or group of clinical signs.

The terms “upper motor neuron” and “lower motor neuron” are commonly used, however, in clinical veterinary neurology to describe a patient’s ***clinical signs*** as “UMN” or “LMN” in character which is an expansion of the original scope of usage for these terms. In a clinical setting when describing patient abnormalities, there appears to be an implied understanding that sensory neurons are likely affected in many diseases that concurrently affect motor neurons. This mixed sensory/motor impairment is common with diseases that affect structural elements of the nervous system at a macroscopic level as these disease processes are not selective for a specific type of neuron. Given that an equal, if not greater, number of disease processes either exclusively or concurrently involve nerves transmitting sensory-related information, it seems medically inaccurate to use the terms “upper motor neuron” and “lower motor neuron” to describe ***clinical signs*** associated with, or reflective of, sensory nervous system dysfunction. Given the mixed sensory and motor pathologies are common disease manifestations, using the terms “central” or “peripheral” nervous system signs would be more anatomically and physiologically accurate to describe the clinical expressions of these pathologies (Table 1). This is especially true when the clinical signs predominantly reflect sensory dysfunction such as ataxia and hypermetria (as discussed further here). This author uses these terms (i.e., central and peripheral) when teaching the genesis of these clinical signs which allows students to conceptualize the various spinal pathways (including both sensory and motor) that contribute to neurologic disease expression.

### 2.2. Descriptions of Patient Movement Abnormalities

Subjective evaluation of gait-related movements is an essential component of neurologic evaluation. Clinical evaluation is based on numerous observable features of independent movement. These subjective assessments have been supported through kinematics and kinetics of animals walking (Table 2) [18,19,20,21,22,23,24,25,26,27,28,29,30,31,32,33,34,35,36,37,38,39,40,41,42,43]. Simplistically, there is an anti-gravity support action (stance or standing) primarily resulting in limb extension and a stepping action primarily executed through coordinated limb flexion. Additionally, there is a complex balance between muscle function of various agonist and antagonist muscles to execute precise spatial and temporal aspects of the movement. Finally, muscles need to produce an adequate amount of power or strength to perform the various motions associated with locomotion given the counterforce of gravity creating body “weight”.

Relative to movement abnormalities, many terms such as “ataxia”, “hypermetria”, “spasticity”, “lameness”, and “paresis” used to describe movement abnormalities have common-knowledge implicit meanings, but not explicit universally agreed upon objective operational and descriptive definitions. The lack of pathognomonic features in many gaiting abnormalities creates opportunities for individual interpretation, adding further confusion in communicating the actual abnormality expressed by the patient to colleagues.

Correspondingly, denotations of these words are generic and do not include clinically observable signs. Therefore, a proposed descriptive definition of each word is provided here (Table 3). These written descriptives are based on clinical observations in patients with confirmed neurologic or musculoskeletal disorders supplemented with information that has been collected objectively through kinematic and kinetic analysis.

**Table 2:** Defining upper and lower motor neuron usage as clinical descriptors.

Original Conceptualization	Common Use Terminology	Clinical Relevance	Recommended Clinical Descriptor
Motor system abnormality involving the central nervous system pyramidal system	Upper motor neuron (UMN) “signs”	Clinical disease often results in a combination of central sensory and motor dysfunction	“Central Nervous system signs”
Motor system abnormality involving the anterior horn cell and associated nerve	Lower motor neuron (LMN) “signs”	Clinical disease often results in a combination of central sensory and motor dysfunction	“Peripheral Nervous system signs”

**Table 3:** Descriptors of abnormal gait-related movements and proposed definitions.

Medical Term	Proposed Descriptive Definition
	All the following descriptions begin with the phrase “An abnormality of independent movement characterized by”
“Ataxia”	An inconsistent footfall pattern (cadence) coupled with an abnormal and inconsistent step width when contacting the ground (transferring from the swing to stance phase of the gait cycle). The abnormal and inconsistent step width results in abnormal single-tracking and circuitous as compared to a linear direction of travel.
“Dysmetria”	An increase or decrease in the active range of motion of a limb during locomotion relative to the normal curvilinear trajectory of the limb movement.
“Hypermetria”	An increase in the active range of motion of a limb during locomotion relative to the normal curvilinear trajectory of the limb movement. This most commonly results in over flexion of at least one of the limb joints in the abnormal limb.
“Hypometria”	A decrease in the active range of motion of a limb during locomotion relative to the normal curvilinear trajectory of the limb movement. This most commonly results in less flexion of at least one of the limb joints in the abnormal limb and a decreased step length.
“Spasticity”	A persistent extended posture throughout the swing phase of the gait cycle resulting from increased extensor muscle tone in the limb.
“Lame” or “Lameness”	A decreased stance time on the affected limb.
“Paresis”	An inability to maintain normal limb extension when standing often with an increased stance time. The hallmark expression of paresis is standing or moving on the dorsal aspect of the affected paw commonly described as “dragging” of the limb.

### 2.3. “Ataxia” [44,45,46,47,48]

“Ataxia” is a term commonly used to describe a diverse spectrum of clinical signs but without a cogent description of the defining clinical features. This term is historically derived from the Greek “a” (“*lack of*”) and “taxis” (“*order or arrangement*”). The dictionary definition of “ataxia” is “*an inability to coordinate voluntary muscular movements that is symptomatic of some central nervous system disorders and injuries and not due to muscle weakness*” [2,3]. These dictionary definitions, however, provide no objective clinical criteria to use to support the clinical decision to apply these terms to a patient’s movement abnormality.

Ataxia is often described in colloquial terms as “uncoordinated” or “unsteady” and sometimes as a “drunken” gait pattern [43]. Other descriptors include having a “widened base”, “unsteadiness”, “irregularity of steps”, and “lateral veering” [48]. Additionally, ataxia is often described in lay terms as a “loss of or poor balance”. While these terms are not medically specific, they do convey the overall clinical appearance and behavior of animals demonstrating ataxia.

**Figure 1:**
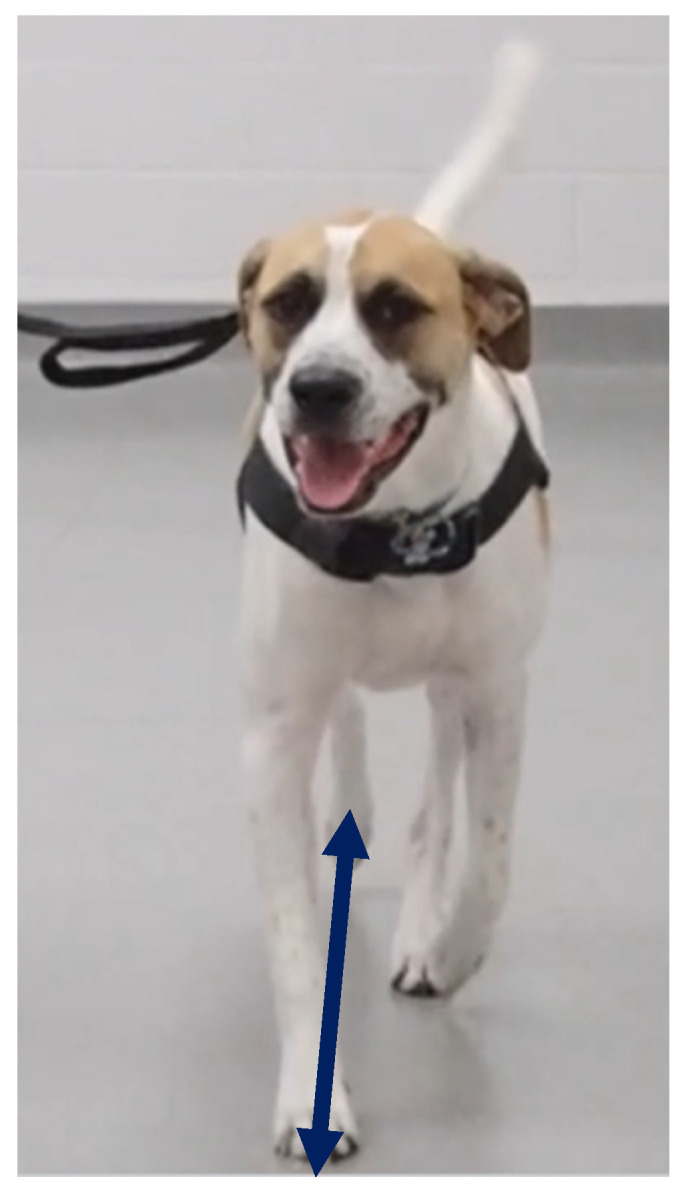
Single-tracking forward movements of a normal dog. The arrow indicates the direction and plane of the dogs foot placement relative to same side thoracic and pelvic limbs.

To identify abnormalities of gait in animals, it is necessary to understand normal gait characteristics for that species [18,19,20,21,22,23,24,25,26,27,28,29,30,31,32,33,34,35,36,37,38,39,40,41,42,43]. When a dog is moving in a forward direction without turning, the optimal line of travel for a dog is linear. A normal dog’s limb movements are stereotypic, and the dog follows a single-tracking gait pattern (Figure 1). The trajectory of a dog’s individual limb movement during stepping follows a curvilinear arc in a median (craniocaudal) plane from ground contact to subsequent ground contact of the same limb. Concurrently, there is limited movement in the mediolateral (“side to side”) plane which maintains the linear forward movement of the dog. The spine is the primary longitudinal axis and the central supportive structure of the body which is an important clinical reference point for motion. The central direction of travel using the longitudinal axis of the spine as a reference is along the median (sagittal) plane of the dog with the limbs traversing in a slightly paramedian (parasagittal) but parallel linear plane. The central axis and center of gravity shift slightly during walking but do so within narrow limits that might not be perceivable during observation of the animal moving in a forward trajectory.

Clinically, ataxia is a deviance from a normal linear movement trajectory observed in numerous neurologic disorders [49,50,51,52,53,54,55,56,57,58,59,60,61,62,63,64,65]. Ataxia is characterized by **abnormal limb trajectory and foot placement** resulting in divergence from a linear forward path. Key features include:

- Abnormal mediolateral deviation of the limb during stepping.
- Irregular or inconsistent foot placement.
- Altered and inconsistent step width, including increased, decreased, or crossing patterns.
- Lateral shifting of the pelvis or trunk.
- Circuitous forward progression.
- Inconsistent footfall timing.

**Figure 2:**
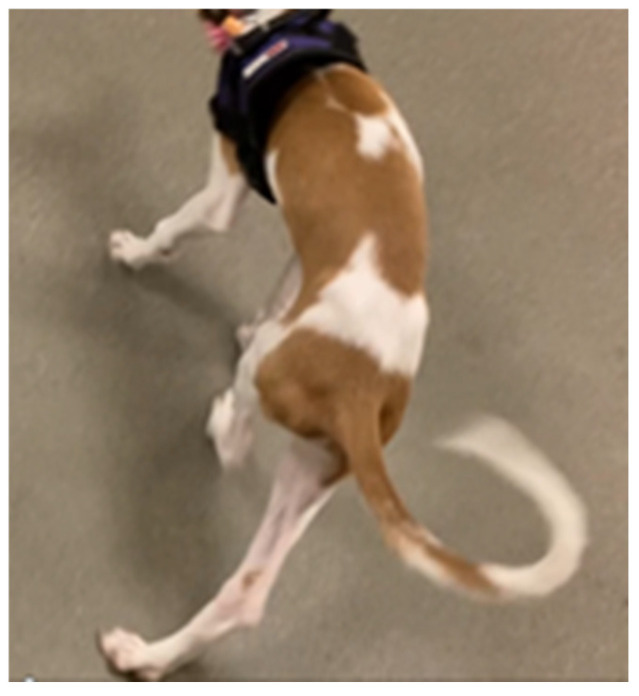
Dog with Ataxia demonstrating abnormal foot placement with ground contact.

**Figure 3:**
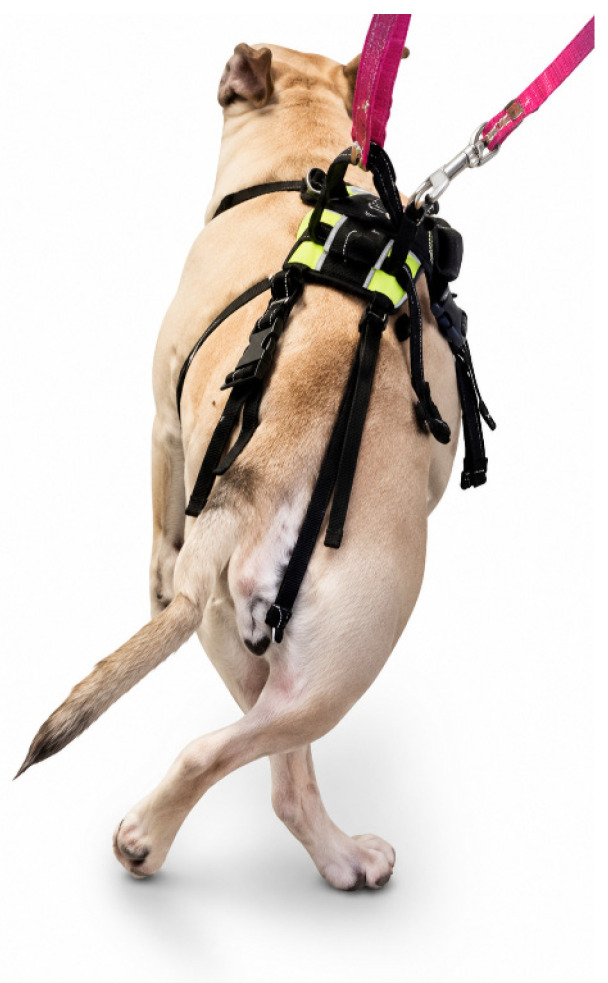
A dog with ataxia demonstrating crossing of the limbs when stepping (AI (ChatGPT 5.5)-enhanced image from an actual patient).

In animals demonstrating ataxia, there is abnormal limb trajectory during gait resulting in abnormal foot placement and shifts in the central axis. This abnormal movement is often accompanied by an abnormal gait cadence and an inconsistent footfall pattern creating an overall “lack of order” to the patient’s movement. Acceleration and deceleration during gait may also follow an irregular pattern. A defining clinical feature of ataxia is deviation from a linear forward trajectory, resulting in abnormal foot placement and inconsistent step width. This is manifested by abnormal foot trajectory and ground contact, often in either a more abducted or adducted position (Figure 2). The alterations in step width of travel ranging from increased to negative (that is, with one limb crossing into the path of another) is a clinical feature of ataxia (Figure 3). Abnormal limb placement during ground contact is a characteristic feature of ataxia and may help distinguish it from other movement abnormalities. Given that this abnormal foot placement position is a deviation from single-tracking, the overall movement pattern is not biomechanically efficient as this foot placement results in the center of gravity shifting from side to side [22].

**Figure 4:**
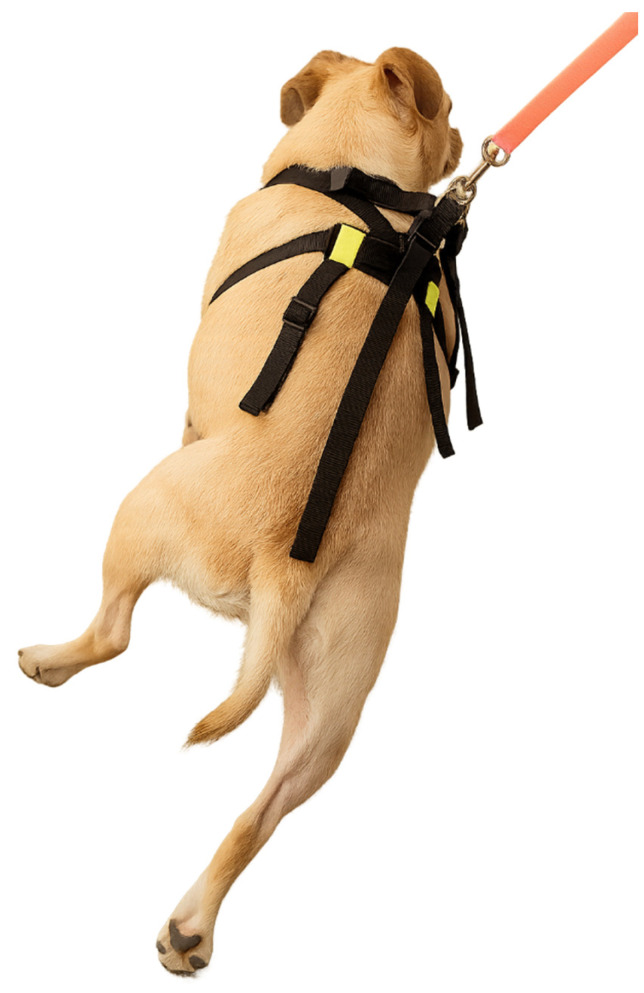
Dog with ataxia falling to one side (AI (ChatGPT 5.5)-enhanced image taken from an actual patient).

When this abnormality occurs in the pelvic limbs, the pelvic region often shifts (most commonly laterally) relative to the longitudinal axis of the body. This is colloquially described as “falling” which may also occur with, and be difficult to distinguish from, paresis (Figure 4). The mediolateral shifting of the patient’s craniocaudal trajectory relative to the central longitudinal axis of the spine results in a circuitous forward body trajectory with repetitive stepping relative to a forward direction.

Ataxia as a unique clinical abnormality is sometimes conflated with other movement abnormalities such as dysmetria or paresis using the umbrella term “ataxia”. The noun “ataxia” is modified by some with adjective terms such as “cerebellar” or “vestibular” [9,66]. These adjectives, however, cannot be applied unless there are additional clinical signs of dysfunction in these neurologic locations. In situations where an accompanying clinical feature is characteristic of dysfunction in specific neurologic regions such as the cerebellum or the vestibular system, it would seem most appropriate to describe a patient with “ataxia” “of cerebellar origin” or “from cerebellar dysfunction” as compared to using the phraseology “cerebellar ataxia”.

Identifying an animal as having ataxia is important given the underlying pathophysiological implications of this clinical abnormality. The fundamental and commonly held assumption is that this movement abnormality results from sensory, particularly proprioceptive, dysfunction. This is why ataxia is often implicitly, and sometimes explicitly, associated with cerebellar, general proprioceptive, or vestibular system abnormalities. Clinical features of any associated motor dysfunction that changes the clinical expression of ataxia as a discrete clinical abnormality should be described separately from the clinical features of ataxia in isolation. When motor dysfunction is concurrently present, the appearance of ataxia may be altered or masked, complicating clinical interpretation and results in a blended clinical manifestation of movement abnormalities. This is likely why ataxia is used by some as an all-encompassing clinical descriptor of mixed sensory/motor nervous system abnormalities.

### 2.4. “Dysmetria” (“Hypermetria” and “Hypometria”)

“Dysmetria” is a general term to indicate improper distance of travel during muscular activity [67,68,69,70]. “Hypermetria” and “hypometria” are sub-types of dysmetria.

The term hypermetria is derived from the concept of “over measurement” from the Greek “hyper” (“*over*”) and “-metria” (“*measurement*”; “*a measurement of*”). The dictionary definition of hypermetria is “*a condition of cerebellar dysfunction in which voluntary muscular movements tend to result in the movement of bodily parts (as the arm and hand) beyond the intended goal*.”

**Figure 5:**
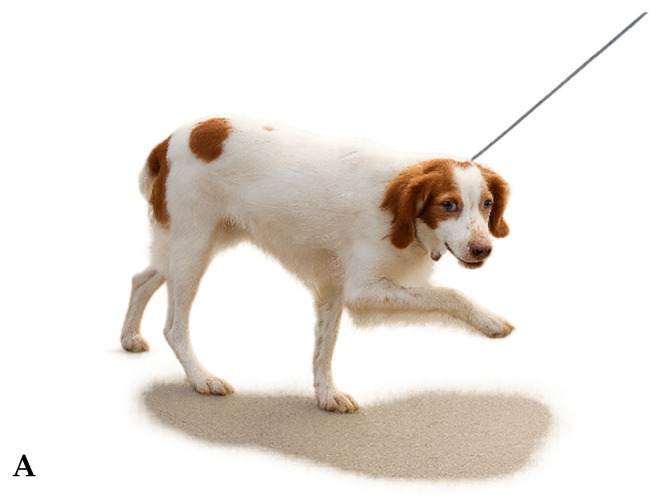

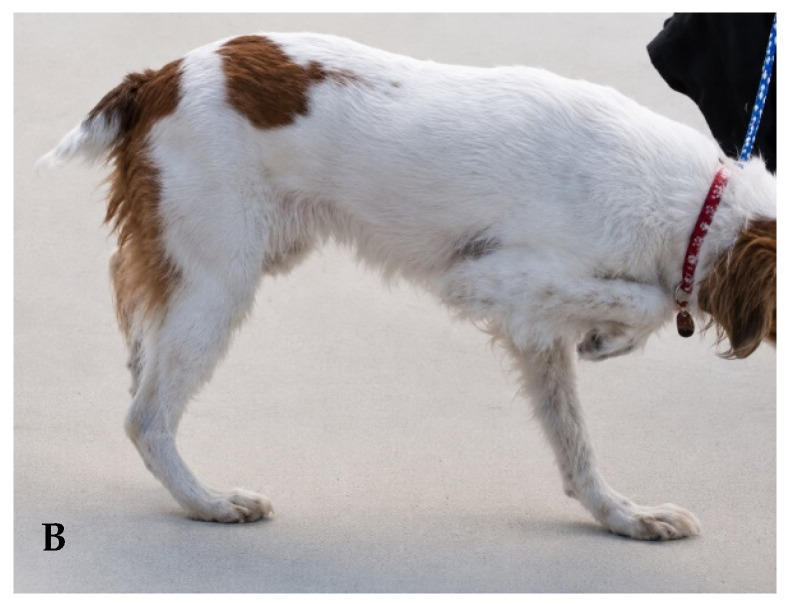
A dog demonstrating hypermetria with different positions of the carpal joint (**A**,**B**). In both instances, however, the amplitude of the swing phase of the gait cycle is excessive (AI (ChatGPT 5.5)-enhanced images taken from an actual patient).

In clinical practice, “hypermetria” is often applied when an animal’s movements are exaggerated or excessive regardless of the specific characteristics of the movement abnormality (Figure 5). When hypermetria is used as a clinical descriptor, this refers to an active range of motion that is greater than normal and commonly manifested as greater flexion in one or more of the limb joints during the swing phase of the gait cycle (Table 3). The nervous system coordinates the swing trajectory of the limb to achieve efficient motion and avoid obstacles in the path of travel. As dogs only use a portion of their available passive range of motion during active range of motion in the gait cycle [71], there is an efficient range of motion to the limb movements relative to the ground surface. This amplitude of flexion keeps the limb in a position to avoid obstacles or contact with the ground surface (preventing friction) while equally inhibiting excessive flexion which creates additional muscle work. During the normal curvilinear swing trajectory of a limb with forward movement on flat ground, for example, the distance a dog’s paw is elevated from the ground surface is approximately 6 cm for Golden retriever dogs [72]. When the sensory (proprioceptive) functions of the nervous system are abnormal, the limb will travel a greater distance than necessary in flight creating the exaggerated appearance in limb movement. With hypermetria in isolation (that is, without ataxia or paresis), the linear trajectory of the foot is normal (normal single-tracking); however, the amplitude of the flight arc of the trajectory is greater than normal (i.e., “over-flexed”). In other instances, “hypermetria” is used to describe combinations of over-flexion and over-extension of one or more of the limb joints in various movement trajectories during the swing/flight phase of the gait cycle.

Hypermetria and other abnormal limb movements are most frequently confused with biomechanical alterations that create either an over-flexed or over-extended limb posture or that prevent the normal trajectory of limb movement during the swing phase of gait. Examples of such conditions include fibrotic myopathies and infraspinatus tendon contracture syndromes [73,74,75]. These muscular abnormalities are more likely to create abnormal limb or foot travel during the gait cycle. As an example, there is circumduction of the thoracic limb trajectory with infraspinatus contracture that results from the external rotation of the humerus at the shoulder joint [73]. With fibrotic myopathy involving the gracilis or semitendinosus muscles, there is a characteristic rapid medial direction change of the tibia/tarsal joint just past the zenith of the swing phase of the step [74,75]. In both myopathic conditions, however, proprioceptive and reflex functions in the affected limb are normal, which supports a non-neurologic cause for the limb movement abnormality.

Hypometria is the opposite of hypermetria, wherein the animal’s limb has less active range of motion during swing. This often results in a slight over-extended appearance of the limb during the swing phase of the gait cycle. The step distance is usually concurrently decreased. These clinical characteristics are not, however, specific for hypometria and are difficult to subjectively separate from paresis. This may contribute to the infrequent documentation of hypometria as a distinct clinical abnormality in dogs.

### 2.5. “Spasticity”

A similar extended limb appearance can occur with “spasticity”. The dictionary definition of spastic is “*relating to, marked by, or affected with spasm (an involuntary and abnormal muscular contraction); characterized by hypertonic muscles*” and/or “*muscular hypertonicity with increased tendon reflexes*.” Spasticity is characterized by **increased muscle tone** and **facilitation of extensor musculature** due to loss of descending inhibitory control [76,77,78,79,80,81,82,83,84,85,86,87,88,89]. Clinically, spasticity produces:

- **Extended limb posture** during stance and swing.
- **“Stilted” or “floating” gait.**
- **Dorsal extension of the digits** during limb advancement (in some but not all cases).
- **Normal or increased step length** (distinguishing it from hypometria).

**Figure 6:**
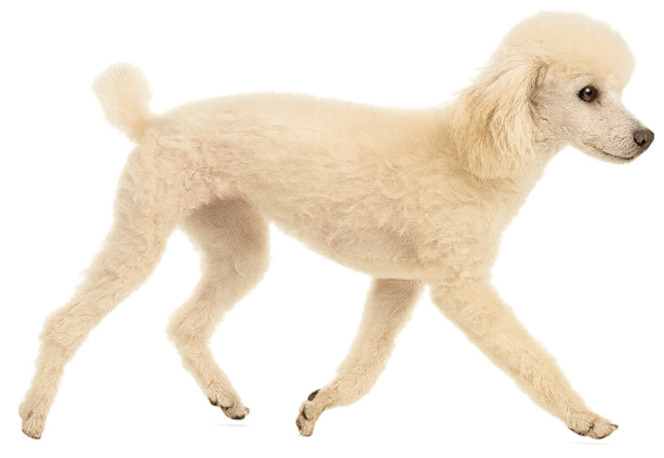
Dog demonstrating spasticity of the right thoracic limb (AI (ChatGPT 5.5)-enhanced image taken from an actual patient).

**Figure 7:**
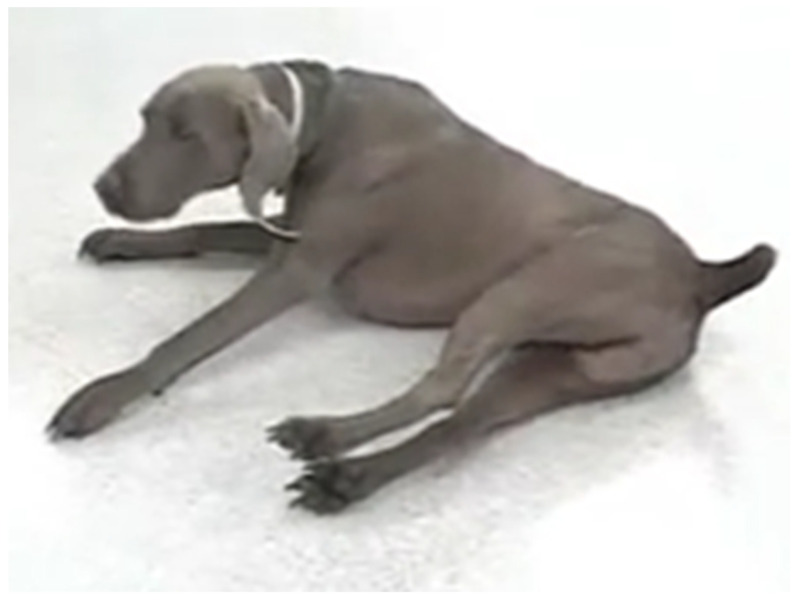
Dog demonstrating spasticity when recumbent.

**Figure 8:**
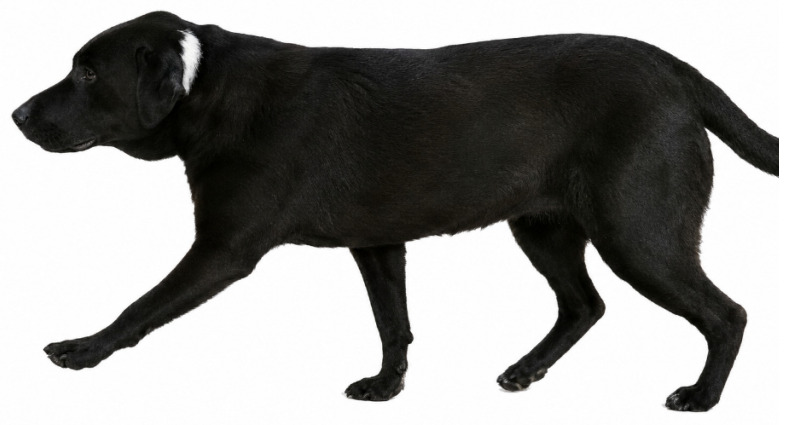
Dog demonstrating slight dorsal extension of the left thoracic limb immediately prior to ground contact (AI (ChatGPT 5.5)-enhanced image taken from an actual patient).

As muscle tone is primarily assessed through palpation, the term “spastic” or “spasticity” is commonly used during subjective gait assessments when an animal’s movements are less “flexible” than expected in either the stance or the swing phase of the gait cycle (Figure 6). Increased muscle tone with spasticity most commonly results in an extensor posture in the affected limb. With central nervous system disease, this posture is believed to result from loss of inhibition on extensor muscle group activity resulting in facilitation of these muscles [75,76,77,78,79,80,81,82,83,84,85,86,87,88,89]. An extended posture can be observed in both the stance and the swing phases of the gait cycle as well as when the animal is in a recumbent position or in the process of standing (Figure 7). During gait, the extended limbs are colloquially described as “floating” or “stilted”. Some animals will have a slight dorsal extension of the digits as the limb is moved toward the ground during the swing phase of the gait cycle (Figure 8). One differentiating feature of spasticity compared to hypometria is that, with spasticity, the stride length is often normal or even increased, whereas with hypometria the stride length is usually shorter.

In addition to having “spasticity”, animals with extensor postures are also colloquially described as “stiff”. The dictionary definition of “stiff” is “*not easily bent: rigid; lacking in suppleness or flexibility*.” This term is most often used when the patient has a more extended posture of the limbs but could also apply to neck posture/movements. “Stiff”, however, is not a medical term and the description of the patient’s posture as “extended” compared to normal would be more appropriate and accurate. Therefore, the term “stiff” should not be used in medical communication given the other more accurate descriptive options.

### 2.6. “Lame” or “Lameness”

The dictionary definition of lame is “*unable or only partially able to use a body part and especially a limb; an abnormality in gait or inability to use one or more limbs*.” This dictionary definition of “lame”, being clinically generic, could be applied to various abnormalities of limb during movement. When used as a common clinical descriptor, however, this term is applied when a patient’s movement has several clinical characteristics epitomized by alterations in the stance and swing phase of the gait cycle (Table 3).

Clinically, lameness is characterized by:

- **Reduced stance time** in the affected limb.
- **Compensatory reduced swing time** in the contralateral limb.
- **Asymmetric inter-step timing** (quick–slow pattern).
- **Head movement changes**, including head elevation during weight transfer onto the affected thoracic limb and lowering of the head during transition to the stance on the normal limb.

**Figure 9:**
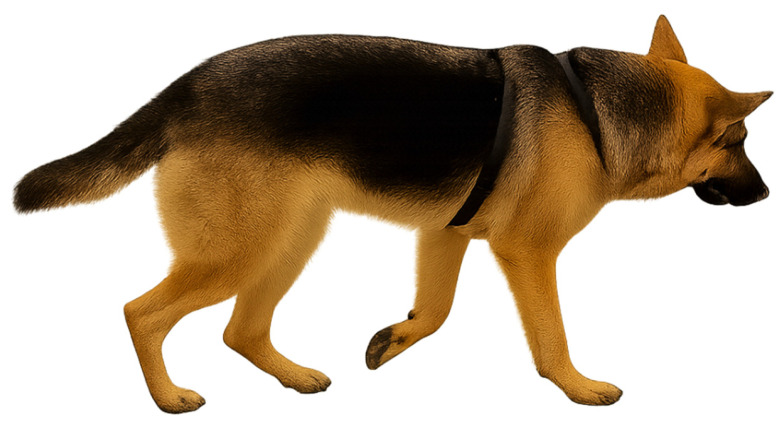
A dog demonstrating short inter-step distances in the pelvic limbs resulting from right pelvic limb pain (AI (ChatGPT 5.5)-enhanced image taken from an actual patient).

If the abnormality is isolated to a single limb, the stance time in the affected limb is shorter than normal with a concurrent decrease in the swing time of the contralateral normal limb [20,29,40,90]. This results in an asymmetric temporal asymmetry between the contralateral pair of limbs (Figure 9). If, for example, the left thoracic limb is affected, the stance time on that limb is shorter (less contact time) compared to the stance time on the opposite normal limb, creating the “quick” phase of opposing limb movement with weight on the affected limb and the “slow” phase with weight on the normal limb. While abnormal for each gait cycle, the movements are stereotypic throughout subsequent gait cycles (e.g., quick followed by slow followed by quick during each successive step) provided that the gait speed does not change. When multiple limbs are affected, the decreased stance time with short inter-step distances in the affected limb(s) remains a consistent clinical feature.

**Figure 10:**
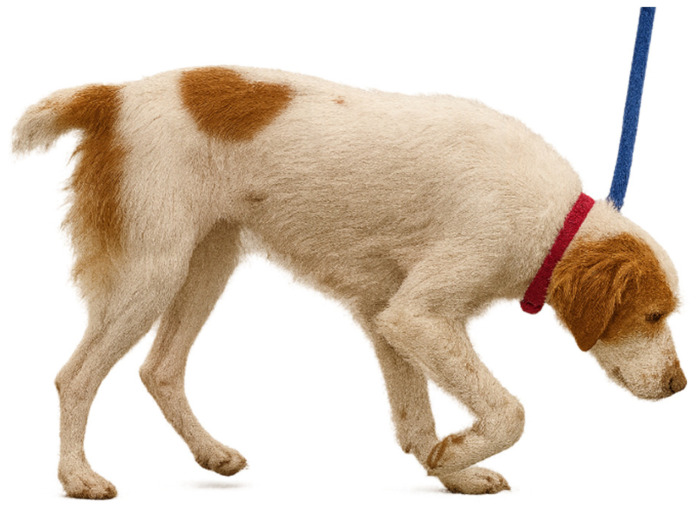
A dog with a right thoracic limb lameness demonstrating a more ventral head and neck position when contacting the ground with the normal left thoracic limb (AI (ChatGPT 5.5)-enhanced image taken from an actual patient).

Unless subtle, there are usually concurrent changes in neck/head movement wherein the animal’s head moves in a ventral direction during the stance phase of the normal limb and in a dorsal direction as weight is shifted from the normal limb to the affected limb immediately before the affected limb enters the stance phase of gait (Figure 10). This is most notable with an abnormal thoracic limb but alterations in head posture can also be observed with pelvic limb lameness.

### 2.7. “Paresis”

The derivation of “paresis” is “*beside*” and “*letting go*” or “*relaxation*”. The dictionary definition of paresis is “*slight or partial paralysis*” with the definition of “paralysis” as “*complete or partial loss of function especially when involving the motion or sensation in a part of the body*.” The pathophysiological definition of paresis is a reduced ability to volitionally activate the spinal motor neurons [91,92,93].

Clinically, paresis appears as:

- Slower (hypokinetic) limb movements.
- Reduced ability to support body weight while standing or walking.
- Sinking or collapsing when weight is placed on the limb.
- Limb rotation around the vertical axis during stance or ground contact.
- Abnormal paw position such as standing or walking on the dorsal aspect of the paw.
- Dragging of the paw, with dorsal surface contacting the ground.
- Abnormal wear of the central toenails (P3 and P4).

Paresis is often used as a general term to describe various movement deficiencies [18,94,95,96,97,98,99,100,101,102,103,104]. In human beings, this can be assessed by instructing the patient to perform various intention movements while assessing the ability to execute these movements. In animals, this assessment is performed by observation of an inability to perform normal or natural movements such as standing and walking. Movements are slower than normal (hypokinesia or bradykinesia) and the animal does not support their body weight normally relative to gravity while standing or moving. With central nervous system disease, there are usually multiple muscle groups that are dysfunctional as compared to a single muscle group. A similar pattern may occur with peripheral nerve disease; however, isolated involvement of muscles innervated by a single affected nerve is also possible.

There are degrees of paresis ranging from mild to severe which are reflected in the resultant clinical signs. When muscles are not activated normally, an abnormality of extensor support to some degree is an observable feature. This dysfunction is described colloquially as “collapsing”, “sinking”, or “dragging”. Animals with neurologic motor dysfunction in a pelvic limb but with some muscle function remaining in the affected pelvic limbs may shift their pelvic area laterally with weight-bearing. When paresis is significant, animals may even collapse to the ground in a sideways posture, contacting the ground with lateral surface of the limbs or pelvic area. This “falling” movement may be confused with ataxia and vice versa. In some animals that are about to stand and support weight during gaiting, as the paretic limb contacts the ground or when the animal’s body weight is placed on the limb, the limb rotates or twists (swivel or twist) around the vertical axis of the paw. In these instances, the caudal aspect of the limb at the tarsal joint region rotates laterally.

When paresis is severe, the animal will not have the ability to stand independently. The limbs may be in an extended or flexed posture based on the degree of extensor tone in the limbs. At this stage, when attempting to make limb movements, the distant limbs are usually more significantly impaired which results in an inability to flex the tarsal or carpal joints when attempting to initiate the swing phase of the gait cycle. This is commonly described as “dragging” of the paw or advancing the limbs on the dorsal aspect of the paw. Some shoulder or hip flexor movements may remain.

Animals with neuromuscular junction or primary muscle disorders can also have an increased flexed posture with weight-bearing but with the limbs in a linear alignment with the longitudinal axis of the spine. If these animals have some motor function remaining, they will often assume a sitting position and/or lie down in a sternal recumbent position. When motor function is significantly impaired to the point of paralysis, these animals commonly assume a lateral recumbent position.

**Figure 11:**
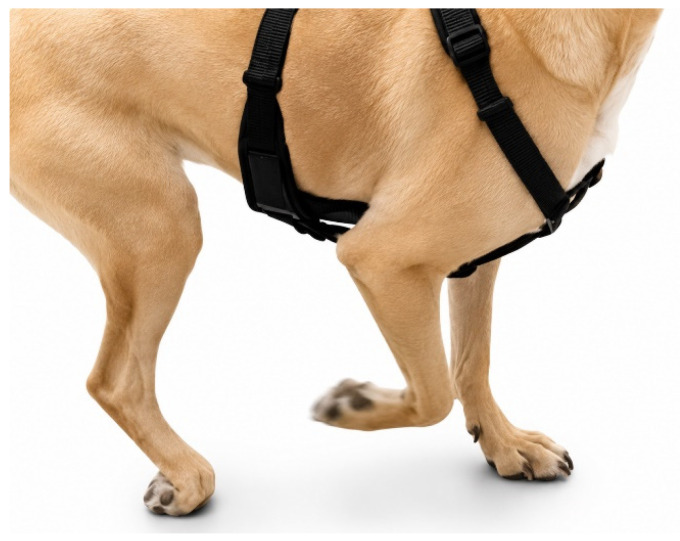
A dog demonstrating paresis by contacting the dorsal aspect of the paw during gaiting (AI (ChatGPT 5.5)-enhanced image taken from an actual patient).

Paresis is most confidently identified when the animal contacts the dorsal surface of the paw with the ground during either the stance or swing phase. (Table 3; Figure 11). In this instance, paretic animals will often stand with the affected paw resting on their dorsal surface. Another clinical clue, usually seen in animals that have motor dysfunction while moving on hard surfaces, is abnormal toenail wear [18]. This abnormal toenail wear involves the two longest toes (P3 and P4). The combination of abnormal toenail wear of P3 and P4 with normal toenail appearance of P2 and P5 is highly suggestive that the animal has paresis. When the animal demonstrates abnormal limb movement without obvious dragging or toenail “scuffing” (“rubbing”), it would seem appropriate to apply the term “paresis” only after demonstrating a neurologic cause for the limb dysfunction through other neurologic examinations (such as proprioceptive positioning or hopping responses).

Paralysis is complete loss of independent movement. Observation for lack of normal muscle movements is the most common method for clinical assessment of paralysis. As paralysis is usually more clinically obvious, there is less confusion when making this assessment as compared to some of the other movement abnormalities such as ataxia or paresis.

## 3. Conclusions

The use of terminology with agreed-upon clinical criteria is essential for accurate neurologic assessment and effective communication in veterinary medicine. Many commonly used descriptors of gait abnormalities—though deeply embedded in clinical practice—lack operational definitions that correspond to observable movement patterns or contemporary neurophysiologic understanding. Movement abnormalities described by terms such as *ataxia*, *hypermetria*, *paresis*, and *lameness* require operational, observation-based definitions to ensure their consistent application. Adoption of universally agreed-upon operational definitions will enhance diagnostic accuracy, improve communication among clinicians and trainees, and support the development of standardized assessment protocols in both clinical and research settings. Continued refinement of neurologic terminology, grounded in clinical observation and objective movement analysis, will strengthen the precision and reproducibility of veterinary neurologic assessment with the goal of improving patient evaluation and management.

## Figures and Tables

**Table 1:** Terminology used to describe aspects of normal gait-related movements in animals.

Medical Term	Clinical Definition
Locomotion	The act of moving from one place to another
Gait	A sequence of foot movements by which an animal moves in a forward direction; a series of stereotypical movements of the limbs of an animal when moving from one location to another
Walk	A gait movement consisting of a sequence of foot placements with a four-beat cadence with at least one limb in contact with the ground at all times; the foot fall sequence is ipsilateral pelvic limb, ipsilateral thoracic limb, contralateral pelvic limb, contralateral thoracic limb
Trot	A gait movement consisting of a sequence of foot placements with a two-beat cadence with two limbs in contact with the ground at all times: the foot fall sequence is repeated simultaneous ipsilateral pelvic limb/contralateral thoracic limb ground contact followed by simultaneous ipsilateral thoracic limb/contralateral pelvic limb ground contact
Gait cycle	Consists of two phases for each limb: a stance phase wherein the limb is in contact with the ground and a “swing” or “flight” phase wherein the limb is lifted off of the ground and advanced in a forward direction; some will use this term to indicate a stride for a single limb where others will use this term to indicate the combination of these phases for each of the four limbs in a quadruped
Step cycle	A step cycle is one-half of a gait cycle of the paired thoracic or pelvic limbs, representing the movement from ground contact of one foot to the ground contact of the contralateral foot within the pair of either the thoracic or pelvic limbs
Step distance	The distance between the thoracic or pelvic pair of limbs when both limbs are in contact with the ground
Stride	The distance traveled during one gait cycle within the gait cycle of a single limb from ground contact to the next ground contact of that individual limb
Active range of motion	The degree of angulation changes of a joint from maximum flexion to maximum extension during the gait cycle
Posture	The position of the body or a body part at any time point during standing and walking
Static posture	The position of the body when not in motion
Dynamic posture	The position of the body when in motion
Velocity of movement	The speed of movement relative to the distance traveled during the movement
Stance	The phase of the gait cycle where the limb is in contact with the ground and the limb joint angles are relatively extended within an active range of motion
Swing	The phase of the gait cycle where the limb is not in contact with the ground and the limb joint angles are relatively flexed within active range of motion
Neutral position	The midpoint of active range of motion angulation of the limb between maximum flexion and maximum extension
Extension	Within the joint angulation of active range of motion, when one or more of the joint angles are greater than the neutral active range of motion angulation
Flexion	Within the joint angulation of active range of motion, when one or more of the joint angles are less than the neutral active range of motion angulation

## Data Availability

Additional videos available upon request from the author.
